# Complicated Pediatric Pneumonia With *Eikenella* Caused by Foreign Body Aspiration: Institutional and Literature Review

**DOI:** 10.1155/crdi/2857930

**Published:** 2025-08-11

**Authors:** Leon Joseph, Marc Assous, Karin Dreifuss, Shmuel Goldberg, Elie Picard

**Affiliations:** ^1^Pediatric Pulmonology Unit, Shaare Zedek Medical Center, Affiliated With the Hebrew University School of Medicine, Jerusalem, Israel; ^2^Microbiology Laboratory, Shaare Zedek Medical Center, Affiliated With the Hebrew University School of Medicine, Jerusalem, Israel

**Keywords:** *Eikenella corrodens*, empyema pleural, foreign body, pleural effusion, pneumonia

## Abstract

*Eikenella corrodens* is a commensal bacterium of the buccal cavity and rarely causes lower respiratory infections in healthy children. Two young patients with persistent pleuropneumonia caused by *E. corrodens* are presented. In both cases, an obstructing endobronchial foreign body was found. Removal of the foreign bodies allowed complete recovery. A local and literature review demonstrated *E. corrodens* causing empyema in only four other cases, all with comorbidities. We conclude that in cases of persistent pneumonia in healthy children due to *Eikenella corrodens*, an aspirated foreign body should be suspected.

## 1. Introduction

Pneumonia with pleural effusion (PPE) accounts for 1.3% of all pediatric hospitalizations in our institution [1] and has a worldwide population incidence ranging from 2 to 18 per 100,000 [2–6]. In children, outcomes are generally good, with minimal long-term sequelae [7] whether a conservative approach is used [8] or a more aggressive approach including thoracocentesis, intrapleural drain, or thoracoscopic surgery [9]. Clinical practice differs as to the appropriate follow-up required after an episode of PPE. A review of 100 children, primarily treated medically, found that the median time to having a normal or near-normal chest X-ray after PPE was 8 weeks but ranged from 3 to 20 weeks [10]. There are no clear guidelines to determine when to initiate further investigation for an unusual or prolonged clinical picture. A potential reason for prolonged symptoms is the presence of an endobronchial foreign body, even in the absence of a typical story. This category has been termed “occult presentation of foreign body aspiration (FBA)” and is defined as those without a history of choking or sudden onset cough and no typical clinical or radiological findings [11].

*Eikenella* species are typically commensurate to the oral cavity as well as the gastrointestinal and genitourinary tracts. They are fastidious, facultative, anaerobic Gram-negative bacilli [12]. They have been described as the cause of various infections in adults, including in the head and neck, joints, heart valves, and others [11]. Pulmonary infections are rare, with few case reports [13, 14]. *Eikenella* species cause infections far less commonly in children, mostly in the head, neck, and endocardium [15], including in recent case reports [16, 17]. Few cases of pediatric *Eikenella* respiratory infections have been described [10].

We report 2 cases of *Eikenella corrodens* (*E. corrodens*) PPE in previously healthy children who had prolonged symptoms, and in both cases an endobronchial foreign body was found. We subsequently reviewed all cases of *E. corrodens* in our institution. The institutional review board approved the study and waived the need for individual consent. The parents of both case reports gave explicit consent for anonymous details and images to be published.

## 2. Case Reports

**Figure 1:**
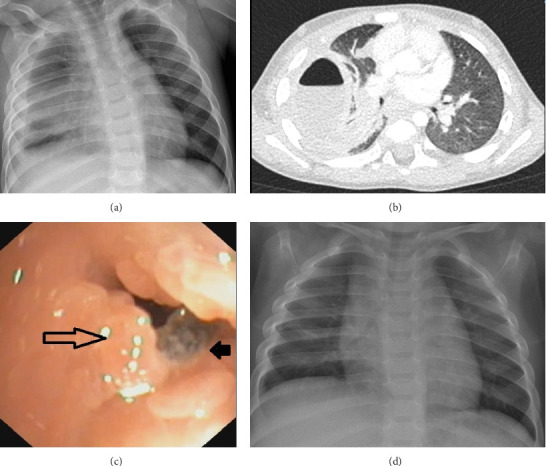
(a) The chest plain radiogram on admission for the first case showing a right lower and middle lobe infiltrate with a pleural effusion. (b) The CT scan for the first case after readmission demonstrating a large, thick-walled effusion on the right with an air-fluid level and an adjacent infiltrate in the right lower lobe. (c) The image from the bronchoscopy in the first case. The black arrow points to the foreign body, and the open arrow shows the granuloma. (d) The chest plain radiogram for the first case after removal of the foreign body and subsequent recovery.

Case 1: A previously healthy 14-month-old presented to the emergency room because of a 6-day history of fever, cough, and lethargy despite 3 days of oral amoxicillin for suspected right-sided pneumonia. On admission, he had high fever, tachycardia, and relative hypoxia (oxygen saturation of 92% in room air). On auscultation, there was reduced air entry at the right lung base. White blood cells were raised at 23,400/L, of which 76% were neutrophils. A chest radiograph showed a large right lower and middle lobe infiltrate with a pleural effusion (Figure 1(a)). Treatment with intravenous cefuroxime was started, an intercostal drain was inserted, and 250 mL of thick purulent fluid was removed from the pleural cavity. *Streptococcus* intermedius and *E. corrodens* grew from the pleural fluid, and the antibiotic treatment was switched to ampicillin according to the sensitivity analysis. His clinical status improved, the intercostal drain was removed, and he was discharged home on oral amoxicillin. Three days later, he was readmitted with a high fever, and a chest X-ray revealed a right lower lung infiltrate with pleural effusion. A pleurocentesis was performed, and a culture of the fluid showed a growth of *E. corrodens* and Prevotella oris. Ceftriaxone was started intravenously, but his fever persisted. A CT scan revealed a large, thick-walled effusion on the right with an air-fluid level and an adjacent infiltrate in the right lower lobe (Figure 1(b)). Because of the persistent findings and the prolonged fever, a flexible bronchoscopy was performed. It revealed a foreign body with significant granulomatous tissue, lodged in the entrance of the basal segments of the right lower lobe (Figure 1(c)). He was started on systemic corticosteroids to reduce the granuloma. Two days later, he had severe bouts of coughing which resolved spontaneously. A subsequent rigid bronchoscopy, performed 3 days after the onset of the steroid treatment, did not show any evidence of foreign body. We assumed that he expectorated the foreign body prior to his rigid bronchoscopy. He made a full clinical recovery, and his follow-up chest radiography was normal (Figure 1(d)).

**Figure 2:**
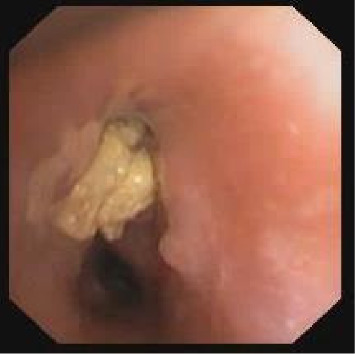
Organic foreign body with granulomatous reaction obstructing the left upper lobe bronchus.

Case 2: A 2-year-old was hospitalized with a history of cough, fever, and lethargy. Physical examination demonstrated reduced air entry throughout the left lung, and a chest radiograph demonstrated a large left-sided infiltrate with effusion. She had a leukocytosis of 62,000/L with 84% neutrophils and a CRP of 27 mg/dL. Cefuroxime was started intravenously. An intercostal drain was inserted, and 700 mL of purulent fluid was drained. *E. corrodens*, sensitive to both second-generation cephalosporins and co-amoxiclav, was isolated from the pleural fluid. After 7 days, the drain was removed, and she was discharged when she was afebrile on co-amoxiclav. At follow-up after 4 weeks, there were persistent crackles on auscultation and reduced air entry on the left. On plain chest X-ray, there was hyperinflation of the left lung. A bronchoscopy was performed, and a piece of organic material with significant granulomatous reaction was found and removed from her left upper lobe (Figure 2). She was treated with Augmentin and systemic corticosteroids, and her follow-up clinical status and chest radiography returned to normal.

## 3. Discussion

We reported 2 cases of complicated pneumonia due to *E. corrodens* that resolved after the removal of a previously unsuspected foreign body from the ipsilateral proximal airway. In both cases, the foreign body was not initially suspected because there was no story of choking. Liu et al. [11] reviewed occult FBA and demonstrated an average time to diagnosis of 3.7 months including a range of hospital stays of 1–18 days until diagnostic fiber optic bronchoscopy. A review from Zhong et al. [18] looking at lower respiratory tract infections secondary to FBA, concluded that age under 2 years, nonsmooth, organic matter retained longer than 7 days are risk factors for infection. Neither study reported any unique bacteriology. Haddadi et al. [19] found that 37 out of 74 children with FBA had secondary pneumonia without specific bacteria identified. Interestingly, in a separate report, 2 children with *E. corrodens* brain abscess were described concomitantly with airway FBA [20]. One child had persistent pulmonary infiltrates without PPE, secondary to an endobronchial foreign body, and the second had a nonobstructing, penetrating airway foreign body (metal needle).

To assess how frequently *Eikenella* species are isolated from the airways, we retrospectively reviewed all cases where *E. corrodens* was isolated in all ages in our tertiary referral center between 2010 and 2021. A total of 146 isolates of *E. corrodens* were found in 77 adults and 44 children. Among the children, 2 patients had a positive culture from pleural fluid (the children described above) and 1 from sputum, a child on chronic hemodialysis who was ventilated because of severe cardiomyopathy. Among the other children, *E. corrodens* grew from the upper airway (21 cases), the blood (1 case), the peritoneum (6 cases), wound swabs (12 cases), and the pericardium (1 case). Among the adults, only 2 patients had a positive culture from pleural fluid and 1 from sputum; all had significant comorbidities.

*E. corrodens* rarely causes PPE in childhood. A case of *E. corrodens* was reported as a cause of necrotizing pneumonia with pleurocutaneous fistula in a child with severe neurological damage and recurrent aspiration [21]. A further case of *E. corrodens* pleuropneumonia was reported in a child with Down's syndrome [22]. St John et al. described 2 children with PPE due to *E. corrodens*. One child had the developmental delay and a clear history of recurrent aspiration pneumonia, and one was previously healthy but had persistent poor air entry and an elevated right hemidiaphragm at the last follow-up [23]. Paul and Patel described 2 further cases of children with *E. corrodens* in tracheal aspirates. One had the pervasive developmental delay, and the other child had cardiomyopathy [12].  Table 1 summarizes the cases of pneumonia due to *E. corrodens* reported in the pediatric literature.

In view of this collective experience, it seems that a positive culture of *E. corrodens* from the respiratory tract is most likely only from children with significant comorbidities. If it is found in a previously healthy child, it should suggest the presence of a foreign body in the airways. Most probably, *E. corrodens* in the oral secretions [24] entered the airways with the foreign body, causing an airway infection with a potential secondary pleuropneumonia. Foreign bodies have been previously described to facilitate the spread of *E. corrodens* infections that originated in the mouth [17, 25, 26].

A limitation of this study is that it is not our standard practice to send the foreign bodies for culture, which could have provided a stronger causative link with the infection. However, the foreign body was removed after a long course of sensitive antibiotics, reducing the likelihood of isolating the bacteria. In addition, *E. corrodens* has been isolated from the mouths of 70% of healthy subjects [27]. The repeated isolation of a bacterium rarely found in pediatric empyema but commonly found in oral secretions led to our recommendation regarding heightened vigilance looking for an inhaled foreign body.

In summary, in a previously healthy child with complicated pneumonia due to *E. corrodens*, a diagnosis of FBA should be suspected. In these cases, lack of adequate response to antibiotic therapy requires early bronchoscopy.

## Figures and Tables

**Table 1:** Cases of respiratory infection due to *E. corrodens* reported in the pediatric literature.

Site of culture	Sex	Age (years)	Comorbid disease	Reference
Empyema	M	0.5	Down's syndrome	[21]
Empyema	F	16	Herpes encephalitis brain damage	[22]
Empyema	M	11	Severe birth asphyxia	[23]
Empyema	M	14	Raised R hemidiaphragm	[12]
Tracheal aspirate	F	7	Mental retardation	[12]
Tracheal aspirate	F	1	Cardiomyopathy	[12]
Empyema	M	1	Foreign body aspiration	Present review
Empyema	F	2	Foreign body aspiration	Present review
Tracheal aspirate	F	13	End-stage renal failure—mechanical ventilation	Present review

## Data Availability

Data sharing is not applicable to this article as no new data were created or analyzed in this study.
